# Strategies for a Rational Use of Opioids in Critical Care Settings

**DOI:** 10.3390/jcm15031039

**Published:** 2026-01-28

**Authors:** Giovanni Misseri, Matteo Piattoli, Alice Mirasola, Lorenzo Guarrera, Carla Evangelista, Giuseppe Cuttone, Luigi La Via, Cesare Gregoretti

**Affiliations:** 1 Department of Anaesthesia and Intensive Care, Fondazione Istituto “G.Giglio” Cefalù, 90015 Palermo, Italyc.gregoretti@gmail.com (C.G.); 2 School of Medicine, Saint Camillus International University of Health and Medical Sciences “UniCamillus”, 00131 Rome, Italy; m.piattoli91@gmail.com; 3 Umberto I Policlinico di Roma, 00161 Rome, Italy; 4 Department of Anaesthesia, San Giovanni di Dio Hospital, 92100 Agrigento, Italy; 5 Anesthesia and Intensive Care Unit, ‘Abele Ajello’ Hospital, ASP Trapani, 91026 Mazara del Vallo, Italy; giuseppe.cuttone@hotmail.it; 6 Department of General Surgery and Medical Surgical Specialties, University of Catania, 95124 Catania, Italy; luigilavia7@gmail.com

**Keywords:** analgosedation, analgesia, intensive care, opioids, opioid stewardship, pain, sedation

## Abstract

Opioids play a central role in pain management and sedation in Intensive Care Units (ICUs), where critically ill patients frequently experience moderate-to-severe pain due to illness and invasive procedures or devices. Uncontrolled pain exacerbates stress responses, contributing to clinical deterioration and adverse outcomes. Although analgesics and sedatives can mitigate these effects, their use must be carefully individualized to avoid complications such as delirium, prolonged mechanical ventilation, and increased mortality. Evidence now shows that excessive or poorly controlled analgosedation can prolong ICU length of stay and delay recovery. Current guidelines recommend opioids as first-line agents for severe acute pain in the ICU, preferably within a multimodal analgesia framework to optimize pain control while minimizing adverse effects. Opioids are also essential for improving tolerance to invasive and noninvasive mechanical ventilation. Modern ICU practice emphasizes an analgesia-first or “analgosedation” strategy, prioritizing pain control with intravenous opioids before adding sedatives. This approach aims to achieve light sedation, reduce ventilator days, and improve overall outcomes. Commonly used opioids include fentanyl, morphine, hydromorphone, sufentanil, and remifentanil, with short-acting agents favored when rapid titration is required. Our narrative review aims to evaluate the clinical impact of opioid use in critically ill patients, including post-ICU outcomes, and to explore the role of opioid stewardship in optimizing patient care.

## 1. Introduction

Opioids in Intensive Care Units (ICUs) are used to provide effective analgesia and sedation for critically ill patients, who are commonly affected by moderate-to-severe pain due to underlying illness, invasive procedures, and the presence of indwelling devices [1]. Unrelieved pain is responsible for the exacerbation of physiological stress responses, thus leading to adverse outcomes and patients’ deterioration [2]. The administration of analgesics and sedatives can mitigate these responses. However, it requires an individualized approach, balancing the pharmacological deleterious side effects such as delirium, fatigue, failure of mechanical ventilation weaning, and increased mortality [1,3]. It was once believed that critically ill patients benefited from deep analgesic sedation, allowing the disease to run its course and resolve with adequate organ support and targeted therapies. However, it is now clearly established that the uncontrolled application of analgesic sedation protocols can prolong ICU-length of stay and therefore impair clinical improvement [1,3,4].

The Society of Critical Care Medicine recommends opioids as the primary pharmacologic therapy for pain management in the ICU, particularly for patients with severe, acute nociceptive pain, and to minimize sedative use and improve outcomes such as ventilator-free days [3]. The American College of Surgeons [5] similarly endorses opioids as first-line agents for severe pain in the ICU, within a multimodal analgesia approach to optimize pain control and minimize opioid-related adverse effects.

Opioids are not only indicated for procedural pain, but also to facilitate tolerance while on mechanical invasive and noninvasive ventilation [6]. Sedation with opioids is primarily used as part of an analgesia-first (analgosedation) strategy, where intravenous opioids are administered to manage pain and provide light sedation. This approach prioritizes pain control before the use of traditional sedatives, aiming to minimize the depth and duration of sedation, reduce ventilator days [7], and improve patient outcomes [1,8].

Opioids are favored due to their rapid onset, titratability, and efficacy in both communicative and non-communicative patients. Fentanyl, morphine, hydromorphone, sufentanil, and remifentanil are commonly used for both analgesia and sedation in the ICU. Short-acting agents like remifentanil are favored when rapid titration and doses adjustment are needed [8]. Light sedation and validated pain and sedation scales to guide opioid dosing and titration have been recommended to minimize adverse effects and the risk of developing dependence [3]. Although analgosedation protocols have demonstrated benefits including shorter duration of mechanical ventilation, lighter sedation levels, and reduced use of continuous sedative infusions [6], opioids can cause dose-dependent side effects, including oversedation, respiratory depression, and increased risk of delirium, particularly when combined with benzodiazepines and especially in elderly patients [2]. Therefore, multimodal analgesia and nonpharmacologic interventions are recommended to further reduce opioid and sedative requirements [4]. Considering reference clinical studies on the use of opioids in ICU, the purpose of our narrative review is to evaluate their clinical impact in the population of critically ill patients, even after their discharge. For this purpose, we will introduce the concept of opioid stewardship, while analyzing the pros and cons of opioid use, and how rationalizing their administration to critically ill patients might positively affect clinical outcomes.

### Methods

This narrative review was conducted to summarize and critically appraise the current and updated evidence regarding the rational use of opioids in adult ICUs, with particular attention to indications, pharmacokinetics, and pharmacodynamics alterations in critical illness, safety, opioid-sparing approaches, and multimodal analgosedation strategies. For the purpose of this review, a comprehensive literature search was performed using electronic databases (PubMed/MEDLINE, Embase, Scopus). The search covered publications from database inception to December 2025. Keywords and Medical Subject Heading (MeSH) were combined using Boolean operators, and included terms related to intensive care (“intensive care unit”, “intensive care”, “ICU”), opioid drugs (“opioids”, “morphine”, “remifentanil”, “sufentanil”, “fentanyl”, “alfentanil”, “hydromorphone”, “methadone”, “analgesics, opioid”), and rational use concepts and terms (“analgesia”, “sedation”, “analgosedation”, “opioid stewardship”, “opioid-sparing”, “adverse effects”, “tolerance”, “withdrawal”, “delirium”). The search was supplemented by manual screening of the relevant literature and major international guidelines on the matter. We considered only articles published in English. Eligible publications included: randomized controlled trials, observational studies, systematic reviews and meta-analyses, clinical guidelines, and expert consensus statements addressing opioid use in adult ICU patients. We excluded studies focusing only on the pediatric population, anesthesia practice, palliative care settings, chronic outpatient settings (e.g., oncological patients), unless these provided data directly related to the ICU context. Article selection was based on relevance to the topic and methodological quality, as assessed by the authors. Given the narrative nature of this review, no formal risk-of-bias assessment or quantitative synthesis was performed. Instead, findings were qualitatively synthesized and organized into thematic areas (pharmacology and pharmacokinetics/pharmacodynamics alterations of opioids in adult critically ill patients, assessment of pain and sedation in ICU, opioid stewardship in ICU, rational opioids section in ICU, multimodal and opioid-sparing strategies, procedural pain, side effects). This narrative approach was chosen to integrate evidence from heterogeneous sources and provide a clinically oriented overview aimed at supporting a rational, safe, and personalized patient-centered approach to opioid use in intensive care practice.

## 2. Pharmacology and Pharmacokinetics/Pharmacodynamics Alterations in Adult Critically Ill Patients

Critical illness results in profound changes to drug pharmacokinetics (PKs) and pharmacodynamics (PDs), thus requiring a higher degree of individualization of drug doses to achieve the appropriate therapeutic target [9]. Understanding these changes might help to improve therapeutic success and limit adverse events related to drug administration. When considering critically ill patients admitted in ICUs, it is important to keep in mind that the heterogeneity of patients population, the pharmacotherapy complexities, the pathophysiologic changes altering metabolism and the potential organ support strategies (such as mechanical ventilation, renal replacement therapy-RRT, extracorporeal membrane oxygenation-ECMO), are the major contributors to inter- and intra-patient variability in drug’s volume distribution (Vd), metabolism, excretion and clearance (Cl) [10]. Haemodynamic instability and cardiac output impairment might reduce hepatic blood flow and interfere with drug metabolism and excretion. In addition, the use of inotropes or vasopressors alters peripheral blood flow, thus affecting drug distribution and metabolism. Drug concentrations may be altered by changes in Vd and binding protein levels, such as decreased serum albumin concentration. Acute kidney injury (AKI) alters drug Cl and affects drug metabolism by a reduction in CYP3A4.

When opioids are considered, critically ill patients present challenges when titrating infusions of sedatives and analgesics to maintain optimal sedation and pain levels [11]. This is explained by all the metabolic derangements and their potentially significant clinical implications. Tailoring dosing strategies based on PK parameters can improve the precision of opioid administration, thereby achieving the targeted therapeutic outcomes and reducing drug accumulation and side effects. The timely evaluation of sedation and pain levels has been associated with shorter opioid duration of infusion, decreased need for mechanical ventilation, and lower risk of infections. However, it has been estimated that under- or oversedation occurs in up to 75% of patients admitted to ICUs for over 24 h [12], and that severe pain prevalence rate rises to 36% [13].

**Figure 1:**
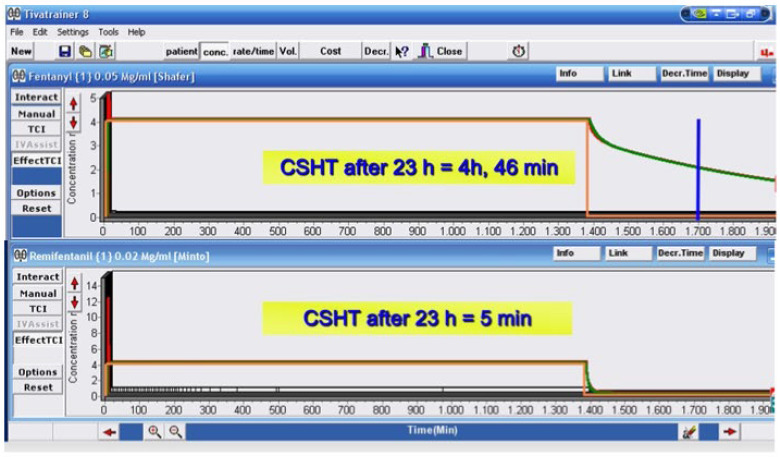
Pharmacokinetic simulation exemplification of Fentanyl and Remifentanil continuous intravenous infusions: context-sensitive half-times (CSHT) after 23 h continuous iv infusion for both opioids are shown (courtesy of Dr. F. Azzeri). CSHT is the time for a 50% decrease in plasma drug concentration, which depends on both drug pharmacokinetics (specifically, distribution and elimination) and its duration of administration (the context). Drugs with high lipid solubility, such as fentanyl, possess a peripheral Vd that is greater than their central compartment’s volume. Therefore, the more prolonged the infusion duration, the more the secondary and tertiary compartments will be saturated. As tissue compartments approach equilibrium with plasma, the 50% decrement time (CSHT) approaches the elimination half-life for a drug; the CSHT approaches but never exceeds the half-life. Remifentanil CSHT is independent of infusion duration (approximately 3 to 5 min) even after prolonged administration. Unlike fentanyl, remifentanil CSHT does not increase over time, allowing easy titration for analgosedation, predictable and rapid awakening (even when used concomitantly with other sedatives), reduced risk of drug accumulation, and easier neurological assessment. Fentanyl has a progressively increasing CSHT due to redistribution into peripheral tissues and context-dependent accumulation. The results are effective analgesia but less predictability when used in continuous IV infusion, higher risk of delayed awakening and oversedation, and need for cautious use when administered in ICU as analgosedation-first strategy. These differences make remifentanil particularly suitable for analgosedation, short-term or rapidly adjustable sedation, daily sedation interruption, and easier weaning.

Despite the widespread use of opioids in critical illness, there is limited data examining the PKs/PDs in critically ill adult patients with prolonged sedatives and opioid infusions (>24 h) to highlight possible altered PK parameters. Mainly used for analgosedation purposes, the PKs of fentanyl, sufentanil, and hydromorphone were observed to be significantly impaired, while morphine, alfentanil, and remifentanil PK impairment resulted in more contained [10]. Compared with noncritically ill patients, the PKs of most opioid infusions in ICUs are usually altered, with T ½ (half-life) being the most affected parameter by critical illness. Although correlations cannot be made, alterations in PKs seem more significant the longer the duration of the infusion. The PKs of drugs that fit a multicompartmental model, such as opioids, are heavily influenced by the length of their infusion. For this reason, the concept of “context sensitive half-time” (CSHT) has been introduced in the case of continuous drug infusions as a more clinically useful term, defined as “the time for the drug plasma concentration to decline by 50% in the context of the duration that the infusion has been running” [14]. This clinically relevant half-life increases following the duration of the infusion until all compartments or body tissues attain equilibrium, reflecting the peripheral compartments saturated with the administered drug. The CSHT eventually plateaus upon reaching the steady state, at which point it is the same as the T ½ and is no longer considered context-sensitive. Figure 1 shows the CSHT of fentanyl and remifentanil after 23 h of continuous infusion (Figure 1).

Drugs with high lipid solubility, such as fentanyl, possess a peripheral Vd that is greater than their central compartment’s volume. Therefore, the more prolonged the infusion duration, the more the secondary and tertiary compartments will be saturated. This causes opioid redistribution, and subsequently, their elimination might occur at a slower rate [15]. The largest study conducted in an ICU evaluated the PKs of fentanyl infusions in 315 patients [16], receiving a mean fentanyl infusion of 1.4 mcg/kg/h for a mean of 58 h. The authors reported that the Vd was 2.2 L/kg and the Cl was 6.3 mL/min/kg. In non-critically ill patients, long-term infusion of fentanyl results in a prolonged T ½ compared with short-term infusion. As fentanyl is highly lipophilic and its Vd is already extensive, marked changes are not expected in critical illness. In addition, its high hepatic extraction ratio suggests that the presence of haemodynamic shock may reduce its elimination [17], and that hepatic dysfunction and cytochrome P4503A4 inhibition (due to commonly used ICU drugs) may potentially reduce fentanyl hepatic Cl [11]. As for sufentanil [18], similar Cl but longer T ½ were found in critically ill patients compared with non-critically ill patients, possibly related to the apparent increase in Vd during the elimination phase. Considering that 90% of sufentanil is bound to plasma proteins, the alterations of their concentrations, which are usually retrieved in critically ill patients, might explain the increase in Vd, thus contributing to the extended T ½ [19].

Morphine, remifentanil, and alfentanil infusions are not associated with altered PKs [10]. Compared with intermittent dosing, the PK parameters of morphine were quite similar when given by continuous infusion in the ICU. However, reduced Cl of morphine predictably correlated with decreasing renal function, where the mean Cl in critically ill patients was up to 10 times less than the Cl reported in noncritically ill patients [20]. Compared with non-ICU infusions, the T ½ and Vd of continuous infusion of remifentanil in ICU were observed to be greater, but Cl was similar [9]. In a study examining the use of remifentanil infusion in 40 critically ill patients with varying degrees of renal impairment [21], the authors retrieved a moderate increase in Vd and a small increase in Cl. The slight variation in Cl could be partially explained by the absence of esterase alterations in critically ill patients. Interestingly, the Vd of remifentanil was increased in patients with renal dysfunction and further increased in subjects requiring renal replacement therapy. The absence of evidence of clinically important PK changes suggests that remifentanil remains the drug of choice due to its rapidly titratable properties. As for alfentanil, no marked changes in PK parameters were reported [10].

## 3. Assessment of Pain and Sedation in ICU: Current Monitoring Standards and Future Directions

The assessment and management of pain in the ICU face many challenges. However, routine pain and sedation evaluations are independently associated with better clinical outcomes, probably because they lead to individualized pain and sedation practices. Mechanical ventilation, use of sedatives, neurological alterations, and critical illness itself induce an impairment in patients’ communication abilities. Often, the uncontrolled use of analgesics is responsible for the dissimulation of symptoms related to life-threatening conditions. On the other hand, insufficient pain control can mask potentially significant illnesses or clinical deterioration. The main consequence of the above observations is that effective pain control cannot prescind from its effective measurement. Intensivists must not assume a linear relationship between illness severity and the pain experienced, as pain has to be considered as an individual and unique sensation, with important interindividual variability.

It is possible to identify three main principles of pain assessment in critically ill patients: (1) identification of the causes of distress and pain, (2) use of validated scales and scores to quantify pain and sedation, (3) integration of vital signs in the assessment of pain and sedation. Traditionally, pain assessment relies on self-reported scales, scored on a range of 1 to 10 (with 10 representing the most severe pain and 0 the least), exemplified by the numerical rating scale (NRS) or its visual version (VAS) [22]. Although recommended by current guidelines, their use is limited to patients able to communicate, be it verbally or non-verbally. For patients with health conditions impairing communication, validated tools such as the Critical Care Pain Observation Tool (CCPOT) and the Behavioral Pain Scale (BPS), are able to objectively assess and characterize pain. Nevertheless, even these are challenged by subjectivity, sensitivity, and inconsistent reliability among different users [1]. Validated sedation and agitation assessment scales commonly used in the ICU include the Richmond Agitation-Sedation Scale (RASS) [23] and the sedation agitation scale [24]. These scales remain the gold standard of sedation monitoring. Alongside advanced monitoring systems, such as processed electroencephalogram systems, usually applied to evaluate anesthesia depth, are being introduced in the ICU to monitor patients’ sedation. However, limited reliability in specific patient populations, interpersonal variability, and artifacts currently limit their widespread use.

While pain might be considered subjective and not efficiently measurable in a sedated patient, nociception can be reliably assessed using several monitoring tools and scores [25]. Although the concept of nociception is still a matter of debate, it can be defined as the pathophysiological responses related to pain. Monitoring nociception would be extremely beneficial in the ICU setting, where pain/nociception is prevalent and patient communication often impaired. The nociception monitoring systems could also be integrated with machine learning algorithms in order to early identify patients experiencing pain, hence allowing a more tailored and timely administration of analgesics. All these monitoring tools rely on detecting the physiological reaction to nociception, namely an increase in sympathetic activity or a corresponding decrease in parasympathetic tone, signaling a shifting in the sympatho-vagal balance. The currently commercialized systems fall into three categories, according to the number of parameters assessed: (1) single-parameter scores (ANI index) [26], skin conductance [27], pupillometry [28], nociceptive flexion reflex [25]; (2) two-parameter scores (qNOX) [29]; (3) multi-parameter scores (nociception level index NOL) [30]. Despite their promising applications, these monitoring tools have been limited to the evaluation of nociception during surgical procedures. In addition, their clinical benefit has not yet been established, since none of these have demonstrated a relevant benefit for their routine clinical use when compared to traditional vital parameters. However, if validated in the ICU, quantification of nociception could promote a personalized approach to pain treatment, thus reducing the incidence of opioid misuse and its related side effects.

## 4. Opioid Stewardship in ICUs

Opioid stewardship is defined as the sum of “coordinated interventions designed to improve, monitor, and evaluate the use of opioids in order to support and protect human health” [31]. The rationale behind its institution is that, according to the United States Center for Disease Control and Prevention (CDC), approximately 105,000 people died from drug overdose in 2023, and nearly 80,000 of those deaths involved opioids (about 76%). This “opioid epidemic” consists of a three-wave shape, each beginning, respectively, in 1999, 2010, and 2013, the causes of which may be related to the rise in the prescription and use of opioids and their semisynthetic derivatives. Recent estimates have confirmed a declining trend of opioid overdose death rate (4%) from 2022 to 2023 [32]. In spring 2023, AHRQ (The Agency for Healthcare Research and Quality) launched the fourth iteration of the MHS (Making Healthcare Safer) reports, identifying opioid stewardship as a high-priority matter [33]. A comprehensive approach to opioid stewardship includes an interdisciplinary and multimodal strategy. However, it is often difficult to counterbalance the excessive opioid use/misuse and the appropriate treatment of pain, especially in specific patient population [34]. Most of the studies on opioid misuse have investigated over-prescription of opioids after surgery, noticing how post-discharge prescription is predicted by pre-discharge use [35], increasing the potential of opioid tolerance and their chronic use [36]. Data concerning discharge opioid prescribing following an ICU stay is more limited: in an observational study [37] involving 120 ICU patients (>65 years of age), 59% of inappropriate medications at hospital discharge were first prescribed during the ICU stay, of which 12% were represented by opioids (started during ICU stay in 73% of cases). A recent retrospective cohort study, including 6764 adults from 21 hospitals affected by acute respiratory failure and receiving mechanical ventilation for more than 24 h, found that opioids administered during mechanical ventilation were associated with opioid prescriptions following hospital discharge [38]: compared with patients who did not receive opioids during mechanical ventilation, a higher daily opioid dose was associated with increased opioid prescriptions in the year after discharge; higher doses of opioids during mechanical ventilation were also associated with a persistent opioid use after hospitalization.

**Figure 2:**
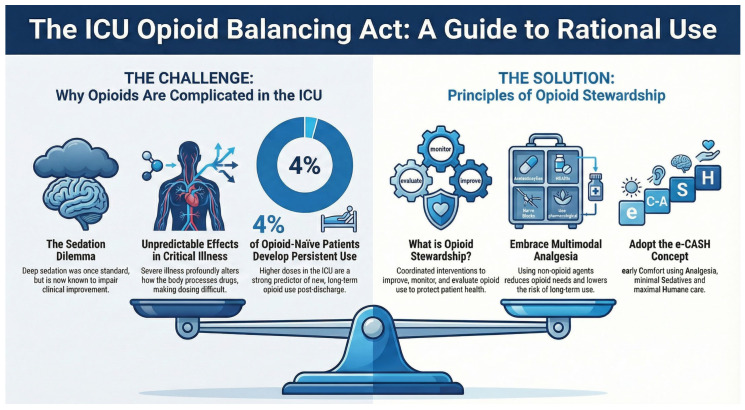
Infographic on opioid rational use in intensive care [4,31,41].

Most of the recommendations pertaining to opioid stewardship protocols have been modeled on the antimicrobial stewardship assumptions [39]. Effective ICU opioid stewardship programs should be founded on multidisciplinary approaches involving physicians, pharmacists, nurses, and pain specialists. This interprofessional collaboration is aimed at implementing evidence-based protocols allowing the appropriate indication of opioid use and its adequate dosage, the attentive monitoring of pain severity, and a reduction in inappropriate prescriptions of analgesics. Multimodal analgesia, compared to opioid-based regimens, is associated with a lower risk of persistent opioid use and may reduce the incidence of chronic pain after ICU discharge [40] (Figure 2). Large cohort studies of ICU survivors demonstrate that early and higher-dose opioid prescriptions—especially potent opioids—are the strongest predictors of new persistent opioid use at six months post-discharge, with approximately 4% of previously opioid-naïve ICU patients developing persistent use; early tapering and multimodal non-opioid strategies are recommended to mitigate this risk [41].

Multimodal analgesia protocols incorporating non-opioid agents (acetaminophen, NSAIDs, gabapentinoids, regional techniques) are demonstrated to be able to consistently reduce inpatient and post-discharge opioid requirements, and are associated with lower rates of long-term opioid therapy in trauma and surgical populations [42,43]. A 2022 pre-post intervention study in orthopedic trauma patients found that a multimodal analgesia regimen (acetaminophen, NSAIDs, gabapentinoids, muscle relaxants, and standardized opioid dosing) led to a significant reduction in long-term opioid use (7.7% vs. 12.0%) compared to opioid-based regimens, with shorter duration and lower daily morphine milligram equivalents prescribed at discharge, without compromising pain control [43]. Similarly, a recent retrospective study in cardiac surgery patients showed that multimodal regimens (including ketamine, dexmedetomidine, and methadone) reduced both intraoperative and predischarge opioid use, with improved pain scores and fewer opioid-related adverse effects compared to opioid-only protocols [44]. A 2025 population-based analysis in coronary artery bypass graft surgery demonstrated a dose–response relationship: increasing the number of non-opioid modalities in multimodal analgesia was associated with stepwise reductions in opioid consumption, postoperative complications, and length of stay [45,46].

There is no evidence that opioid-based regimens improve long-term pain outcomes compared to multimodal or opioid-free approaches; in fact, excessive opioid prescribing is linked to increased adverse events and does not confer superior pain control or patient satisfaction after discharge [44]. Chronic pain after ICU admission remains common, but individualized multimodal pain management and opioid stewardship are advocated to prevent transition to chronic pain and opioid dependence [47,48]. The American Society for Enhanced Recovery and the Perioperative Quality Initiative recommend multimodal analgesia and opioid minimization for both opioid-naïve and chronic users, emphasizing tailored regimens and careful discharge planning to reduce persistent opioid use and chronic pain [49]. While evidence supports the use of multimodal analgesia protocols, direct comparative studies on long-term outcomes and optimal drug combinations in ICU survivors remain limited. Ongoing randomized trials are evaluating standardized multimodal strategies, including combinations of nefopam, tramadol, ketamine, and remifentanil, to further define best practices for opioid minimization and chronic pain prevention in ICU populations [50,51]. Due to the presence of PK/PD alterations in critically ill patients, it is even more important to apply the principles of opioid stewardship programs. In these circumstances, therapeutic drug monitoring (TDM) should be explored [10]. Opioids TDM could guide clinicians on dose initiation and titration to achieve proper PDs and contain opioid misuse and its side effects. In particular, the opioid TDM application in critical care could guide individualized opioid-weaning protocols and, if clinically necessary, pre-discharge prescription. A recent retrospective cohort study [52] examining opioid administration and opioid weaning practices in mechanically ventilated critically ill adults found that 75.8% (n = 240) experienced at least one failed attempt in opioid weaning, and that higher cumulative opioid exposure and prolonged infusion duration were significantly associated with weaning failure. These findings suggest that opioid stewardship protocols, with standardized opioid weaning protocols in ICUs and structured strategies to gradually reduce opioid use, should be implemented and adopted.

Artificial intelligence (AI) might play an increasingly important role in opioid stewardship programs by enabling risk prediction, clinical decision support, and interventions to optimize opioid prescription and personalize pain management strategies [53]. Although AI-based predictive models and screening tools have shown great promise in risk stratification and management of patients receiving opioids [54,55], there is currently no direct evidence regarding the use of AI in opioid stewardship programs for critically ill patients in ICUs. Recent reviews and consensus statements [56,57,58] highlight that AI applications in the ICU have focused primarily on prognostic modeling, early warning systems, workflow optimization, and decision-making processes with limited clinical and real-world impact for therapeutic stewardship tasks. Further studies are needed to prospectively validate, standardize reporting, and implement AI in ICU settings before it can be safely and effectively used for opioid stewardship programs.

## 5. Rational Opioid Selection in ICU Analgosedation Practices

Several studies have identified that early deep sedation in ICU patients is strongly associated with poor long-term clinical outcome, increased mortality [51,59], cognitive decline, and delirium [60] and psychological implications [61]. For these reasons, an integrated and adaptable approach has been suggested to improve patient care and clinical outcome through patient-centered analgosedation strategies (e-CASH: early Comfort using Analgesia, minimal Sedatives and maximal Humane care) [4]. This approach aims to establish an optimal patient comfort using minimal sedation: adequate treatment of pain is the priority, and should use tailored multimodal analgesia practices, designed to minimize the use of opioids. When deciding which opioid should be administered, intensivists should consider the time of onset, planned duration of use, renal or hepatic metabolism alterations, need for mechanical invasive or noninvasive ventilation, and the use of organ support strategies, such as ECMO or RRT. Only a few comparative trials have been performed in the ICU [62]. Opioids such as fentanyl, morphine, hydromorphone, sufentanil, and remifentanil are commonly used for analgosedation in the ICU (Table 1). Remifentanil provides better outcomes than morphine in terms of time at sedation target, use of supplemental sedation, and duration of mechanical ventilation. If compared to fentanyl, remifentanil has revealed equal efficacy in achieving time at target sedation with no difference in extubation times [62]. Hemodynamically unstable patients may benefit from short-acting opioids like fentanyl, sufentanil, or remifentanil due to their favorable PKs and cardiovascular stability. Remifentanil, with organ-independent metabolism, is particularly suitable for patients with multiple organ dysfunction. Regardless of the used drug, sedation and pain should be repeatedly assessed in order to target correct opioid dosing and identify the emergence of adverse events.

## 6. Use of Opioids for the Treatment of Procedural Pain

Procedural pain, which is an extremely common condition in ICUs [63], can be defined as the acute pain occurring in response to diagnostic, therapeutic, or routine care procedures [3]. It should be distinct from other forms of pain, such as disease or surgery-related pain, as it stems directly from the procedural action and is generally fleeting, peaking during or immediately following the procedure itself [3]. Entailing both an emotional and sensory dimension, patients are not only subjected to physical discomfort but also to a deep psychological distress due to the memory of previous painful procedural experiences [64]. Puntillo et al. [63] found that chest tube removal, wound drain removal, and arterial line insertion were the most painful procedures. Together with other procedures, these three can result in a twofold increase in pain intensity from baseline. Factors contributing to more intense procedural pain include the specific type of procedure, an elevated preprocedural pain and emotional distress, a greater preprocedural patient-reported “worst pain” intensity, and the targeted administration of opioids to manage procedural pain. Regarding opioid administration as a risk factor for higher procedural pain intensity [62], there are different underlying possibilities: (1) the overall dose of opioid received may not have been enough to adequately control the procedural pain; (2) the dose of opioid may not have been timely administered to achieve a peak effect in relation to increased pain during the procedure; (3) opioids may have been administered to subjects who had suffered more pain during previous procedures, and (4) opioids may have been given mostly during procedures which were more painful. The Society of Critical Care Medicine recommends the use of fentanyl in critically ill adults, for its rapid onset and short duration, which are ideal PK and PD characteristics in the management of procedural pain [3]. Other opioids as hydromorphone, morphine, and remifentanil, may be considered as alternative options. However, the optimal use of opioids in the control of procedural pain should always consider PKs and PDs features, helping clinicians in selecting the appropriate drug, the right timing of drug administration, and the potential combination of opioids with other analgesics to promote multimodal analgesia strategies.

## 7. Side Effects of Opioids in Critical Illness

Long-term use of opioids may have detrimental effects, with tolerance and physical dependence being the most common [2]. Opioid tolerance, which is characterized by escalating dose requirements to maintain adequate analgesia, may contribute to opioid-induced hyperalgesia. Major trauma, burns, and pediatric patients are at the highest risk of opioid tolerance, due to their need for prolonged mechanical ventilation and higher doses to control pain [65,66]. PK and PK mechanisms might explain the emergence of tolerance and the need for dose escalation in critically ill patients [2]. Cytochrome P-450 inducers increase the clearance of some drugs like methadone, while during the hyperdynamic phase of trauma and sepsis, the enhanced elimination of drugs, such as fentanyl and morphine, could cause the need to achieve higher opioid doses. In addition, inflammation may be responsible for the reduction in opioid efficacy due to cytokine release and altered levels of drug-binding proteins. When considering PDs, the persistence of opioid receptor stimulation leads to an imbalance between pro- and anti-nociceptive cellular signaling pathways, resulting in a reduction in opioid efficacy, increased tolerance, and opioid-induced hyperalgesia. Different strategies should be applied in order to avoid the emergence of opioid tolerance and opioid-induced hyperalgesia: reduction in doses and duration of treatment, daily analgosedation interruptions [67], adequate monitoring and assessment of pain and sedation [4], multimodal analgesic protocols [68] and sequential rotation of opioids [69].

Opioids can also contribute to delirium, poor sleep quality, and unintended sedation. Mistakenly considered inducers of physiological sleep [70,71], opioids dose dependently inhibit sleep, especially SWS and REM sleep [72,73]. The mechanisms causing opioid-induced REM sleep inhibition have been explained by cholinergic neurotransmission, activation of G-protein activity in REM sleep– related nuclei, on the medial pontine reticular formation, and μ receptor–selective inhibition of REM sleep. The emergence of side effects is particularly true in critical illness, where clearance of opioids and their metabolites is influenced by concomitant therapies, comorbidities, haemodynamic alterations, organ and metabolic dysfunction. As far as delirium is concerned, the use of opioid–benzodiazepine combination has been associated with a greater risk in the elderly population admitted to ICUs [3].

In conclusion, the most effective way to address opioid side effects remains their prevention. For this reason, it is prudent to rationalize opioid administration in critically ill patients and use alternative strategies when feasible. In addition, the use of opioids and their prescription should be re-evaluated before ICU discharge and transition to homecare. Opioid stewardship principles should also integrate timely patient access to ICU clinics [74], easing chronic pain re-assessments and opioid use.

**Table 1:** Pharmacokinetics (PK), Pharmacodynamics (PD), initial dosing, and major side effects of key opioids for analgesia and sedation in intensive care (opioid-naïve critically ill patients).

Opioid	Pharmacokinetics (PK)	Pharmacodynamics (PD)/Receptor	Dosage	Major Side Effects/Considerations
**Fentanyl**	**Onset (IV):** 1–2 min [75,76]. **Half-life (IV):** 1–4 h [76]. **Metabolism**: Primarily CYP3A enzymes in the liver via N-dealkylation [75,76]. **Active Metabolites**: None [76]. **Elimination**: Renal [76]. **Accumulation**: Yes, due to distribution into lipid-rich tissues, especially with prolonged infusion or hepatic impairment [75,76].	**Receptor**: μ-receptor agonist [3]. **Effect**: Analgesia, respiratory depression, sedation, gastrointestinal (GI) dysmotility, and pruritus [76].	**IV Push:** 25–50 mcg every 30–60 min PRN [75]. **IV Infusion:** 25–100 mcg/h [75].	**CNS/Resp:** Risk of respiratory depression and sedation [10]. **Cardio**: Causes less histamine release than morphine; preferred in hemodynamically unstable patients or those with renal insufficiency [75]. **Other**: Weakly associated with serotonin syndrome [75].
**Morphine**	**Onset (IV):** 5–10 min [75,76]. **Half-life (IV):** 3–5 h [75]. **Metabolism**: Glucuronidation in the liver (Phase II) [75]. **Active Metabolites**: Morphine-6-glucuronide (active analgesic) and Morphine-3-glucuronide (neuroexcitatory potential) [3,75,76]. **Elimination**: Renal and fecal [76].	**Receptor**: μ-receptor agonist [75]. **Effect**: Analgesia, respiratory depression, sedation [75].	**IV Push:** 2–4 mg every 1–4 h PRN [75]. **IV Infusion:** 1–10 mg/h [75].	**Accumulation**: High risk for accumulation with hepatic or renal impairment due to active metabolites (M6G/M3G) [75]. **Cardio**: May cause hypotension and histamine release [10].
**Hydromorphone**	**Onset (IV):** 5–15 min [75]. **Half-life (IV):** 2–3 h [75]. **Metabolism**: Glucuronidation (Phase II) [75]. **Active Metabolites**: Hydromorphone-3-glucuronide (H3G) (neurotoxic potential) [75]. **Elimination**: Renal [75].	**Receptor**: μ-receptor agonist [75]. **Effect**: Analgesia, respiratory depression, sedation [75].	**IV Push:** 0.2–0.5 mg every 1–3 h PRN [75]. **IV Infusion:** 0.5–2 mg/h [75].	**Accumulation**: Accumulation risk with renal impairment [75]. **Cardio**: Minimal histamine release. PKs are significantly impaired when administered as a continuous infusion in the ICU [10].
**Remifentanil**	**Onset (IV):** 1–3 min [76]. **Half-life (IV):** <1 h (3–10 min) [76]. **Metabolism**: Hydrolysis by plasma and tissue esterases [3,75,76]. **Active Metabolites**: None [76]. **Accumulation**: No [76].	**Receptor**: μ-receptor agonist [75]. **Effect**: Analgesia, sedation [75].	**IV Infusion:** 0.01–0.05 mcg/kg/min [75].	**Toxicity**: No dose adjustment needed for renal impairment. Due to ultra-short half-life, cessation results in rapid loss of analgesia. Associated with increased hyperalgesia upon discontinuation. Can cause significant bradycardia [75].
**Sufentanil**	**Onset (IV):** 1–3 min [75]. **Half-life (IV):** 0.5–2 h [75]. **Metabolism**: Liver (CYP3A4) and enterocytes of the small intestines [75]. **Active Metabolites**: None [75]. **Elimination**: Renal and biliary [75].	**Receptor**: μ-receptor agonist [75]. **Effect**: Analgesia, sedation [75].	(Data for continuous infusion showed rates of 0.8 ± 0.6 mcg/kg/h for up to 20 days) [10].	PKs are significantly impaired when administered as a continuous infusion in the ICU. Accumulation risk exists with hepatic impairment [10]
**Methadone**	**Onset (IV):** 10–20 min. **Half-life (PO):** 8–59 h (mean 35 h) [9,10]. **Metabolism**: Hepatic, primarily CYP3A4, CYP2B6, etc. [9,10]. **Active Metabolites**: None [9,75].	**Receptor**: Full μ-receptor agonist, non-competitive N-methyl-D-aspartate (NMDA) receptor antagonist, and serotonin/norepinephrine reuptake inhibitor [3,75].	(Generally used for maintenance treatment, not acute ICU infusion). **PO:** 5–20 mg (dose varies widely) [3]	**Cardio:** Risk of QT-interval prolongation and Torsades de pointes (TdP) [3]. **CNS:** Serotonin syndrome risk. Long and highly variable half-life [3].

## 8. Conclusions

Growing scientific evidence supports rationalizing the use of opioids in the treatment of critically ill patients admitted to ICUs. Multimodal analgosedation strategies have been demonstrated to be potentially the best choice in reaching optimal clinical outcomes. However, further studies are needed to verify the adequacy of such multimodal regimens in the treatment of critically ill patients. Both pharmacological and non-pharmacological approaches should be integrated to adequately control pain and optimize sedation targets. Short-acting medications, which are characterized by easier titration based on patients’ clinical assessment, are associated with improved short- and long- term outcomes. Opioid use in the ICU has been shaped over the past decades, changing the way intensivists treat patients. Personalized approaches should be adopted, basing the drug choice on comorbidities (e.g., heart failure, transplant recipients, substance abusers) and on the critical illness treated (e.g., patients admitted to ICU after major surgery, trauma, neurological conditions, respiratory failure requiring mechanical ventilation). In this perspective, opioid stewardship programs should be progressively integrated into ICU clinical practice.

## Data Availability

The original contributions presented in the study are included in the article, further inquiries can be directed to the corresponding authors.
